# Cation Composition Influences the Toxicity of Salinity to Freshwater Biota

**DOI:** 10.3390/ijerph20031741

**Published:** 2023-01-18

**Authors:** Cátia Venâncio, Karen Caon, Isabel Lopes

**Affiliations:** Centre for Marine and Environmental Studies (CESAM) and Department of Biology, University of Aveiro, Campus Universitário de Santiago, 3810-193 Aveiro, Portugal

**Keywords:** salinization, ionic composition, magnesium chloride, potassium chloride, calcium chloride

## Abstract

The effects of salinization on freshwater ecosystems have been estimated by testing sodium chloride (NaCl) since it is the most widely used salt as a deicing agent and Na^+^ and Cl^−^ ions are the most representative in seawater composition. However, calcium, magnesium, and/or potassium are starting to be proposed as potential surrogates for NaCl, but for which ecotoxicological effects are less explored. This study aimed to identify (i) the less toxic salt to freshwater biota to be suggested as a safer alternative deicer and (ii) to contribute to the lower tiers of salinity risk assessment frameworks by identifying a more suitable surrogate salt than NaCl. The battery of ecotoxicity assays with five key trophic level species showed that among the tested salts (MgCl_2_, CaCl_2_, and KCl), KCl and CaCl_2_ seemed to induce the highest and lowest toxicity, respectively, compared with NaCl. CaCl_2_ is suggested as a safer alternative for use as a deicer and KCl as a surrogate for the risk assessment of seawater intrusion in coastal regions. These results enrich the salt toxicity database aiming to identify and propose more suitable surrogate salts to predict the effects of salinization to a broader extent.

## 1. Introduction

The salinization of freshwater ecosystems is a worldwide environmental concern, and its causes are many. From the list of various causes, it can be pointed out that the use of thawing salts and seawater intrusion (as a consequence of sea level rise) are the two most relevant. In view of these two scenarios that may lead to freshwater salinization, increased scientific attention has been devoted to assessing the adverse effects of salinization in freshwater ecosystems around the world and at different levels of biological organization, from biochemical to ecosystem levels [1,2,3,4,5,6]. At the individual level, being hyper/iso-osmoregulators, freshwater organisms control osmotic pressure and body ionic composition through several physiological responses. However, above certain salinity levels, these mechanisms may be compromised and become inefficient/insufficient; therefore, an organism’s survival and other fitness-related endpoints such as behavior, feeding, growth, and/or reproduction, may be impaired [2,3,7,8]. These effects at the individual level, in turn, may begin cascade effects at higher levels of biological organization, such as shaping community structure by wiping out the most sensitive species [2,9,10].

Much of the research carried out so far in the context of salinization, whether addressing the use of road deicing or sea level rise, has concentrated efforts in the construction of a solid database regarding sodium chloride (NaCl) ecotoxicity. This choice is related to the fact that the monovalent ions sodium and chloride (Na^+^ and Cl^−^, respectively) are identified as the major contributors in many road salts and in seawater (SW) composition [5,11,12,13,14,15]. In the context of the use of NaCl salt as a deicer or as a major representative of many deicing salts, the use of other salts that are supposedly more environmentally friendly while presenting high efficiency in defrost operations has been recently called for [16,17,18,19]. However, the categorization of other salts as more environmentally friendly still requires further scientific evidence, because, to date, aside from the insipient available empirical ecotoxicological data for some freshwater groups or species (for instance, cnidarians), toxicity data are mostly reported for lethal effects, thus, neglecting responses that express lower levels of toxicity and the toxicity ranking of the salts varies substantially according to the selected study species. For instance, Coldsnow and Relyea [20] found a large differential sensitivity of the Asian clam *Corbicula fluminea* to the three most tested road salts, with the following reported toxicity ranking (descendent order of toxicity): MgCl_2_ (LC_50,8d_ of 1769 mg Cl^−^/L) > CaCl_2_ (LC_50,8d_ of 2235 mg Cl^−^/L) > NaCl (LC_50,8d_ of 10,069 mg Cl^−^/L) [20]. By changing the species and looking at sublethal levels, this toxicity order can change, as shown by the results of Hintz and Relyea [21]. These authors evaluated the growth rate of a salmonid species (*Oncorhynchus mykiss*) when exposed to the same three salts and found the following toxicity ranking: CaCl_2_ > NaCl > MgCl_2_ (this later salt did not induce any effect in salmonid across all tested concentrations). Moreover, the salt NaCl has been suggested as a surrogate of SW within salinity risk assessment frameworks for two major reasons: (i) NaCl has by far a large toxicity dataset, which avoids further animal experimentation, and its availability facilitates the drafting of guidance documents for the derivation of water quality criteria by various government entities (reviewed by Evans and Frick [22]), and (ii) results obtained from NaCl have pinpointed this salt as a worst-case scenario regarding salinization situations as documented by Venâncio et al. [23]. However, the adequacy of other major salts/ions present in SW as a more protective alternative to NaCl has not yet been addressed. The suggestion of other surrogate salts in preliminary risk assessment frameworks would allow for the derivation of more conservative and protective measures for coastal areas at risk.

Ions composing SW or present in deicing salts are diverse (calcium, magnesium, potassium, sodium, chloride, sulfate, carbonate, and bicarbonate [24,25]) and the requirements in which organisms need each specific ion may vary substantially because each ion plays a different physiological role [26,27,28]. As such, each specific ion may also induce significantly different effects when available at concentrations above their equilibrium or homeostatic state [29]. Although conductivities may be reported in the literature allowing later comparison of datasets, different ionic compositions may lead to very different outcomes [30]. Understanding the individual toxicity of different cations may allow the derivation of more reliable ion-specific water quality guidelines [31]. Despite some data being produced over the years on this matter—for instance [6,11,12,32]—the generation of more information with a wider array of species, trophic guilds, and/or endpoints must be pursued to increase the robustness of the aforementioned guidelines.

Hence, within this context, it is important to continue the research to develop tools to understand and characterize the toxicity of the main ions inducing toxicity to freshwater organisms and to further enrich the salt ecotoxicity database. Research should aim to seek out and propose more suitable surrogate salts in order to facilitate prediction of the effects of salinization at a broader extent. This work may contribute substantially by providing data on several different species belonging to different trophic guilds. Accordingly, two specific objectives were established in this study: i) to identify the salt that is less toxic to freshwater biota, to be used as an alternative deicer; and ii) to identify the most suitable surrogate salt for use in lower tiers of salinity-risk assessment frameworks. To achieve these objectives, the toxicity of three salts, differing in their cationic composition (calcium chloride-CaCl_2_, potassium chloride-KCl, and magnesium chloride-MgCl_2_) was assessed at lethal and sublethal levels to several key freshwater species (*Raphidocelis subcapitata*—population growth rate and yield, *Daphnia magna*—survival, somatic growth, feeding rate, *Brachionus calyciflorus*—mortality and reproduction, *Hydra viridissima*—mortality and malformations, and *Danio rerio*—mortality and malformations). The data obtained were then further compared with the extensive dataset available for NaCl (please see Venâncio et al. [23]) to verify if any of the other salts may be used as potential surrogate salt.

## 2. Materials and Methods

### 2.1. Tested Chemicals

The following salts were used to perform the lethal and sublethal toxicity assays: calcium chloride dihydrate (CaCl_2_·2H_2_O), potassium chloride (KCl), and magnesium chloride hexahydrate (MgCl_2_·6H_2_O). All salts were supplied by Merck (Darmstadt, Germany), EMSURE^®^ ACS, reagent grade. The test concentrations of the three salts were prepared by directly dissolving the previously calculated amount of salt in the test medium used for each model species. Only fresh solutions were used to perform the toxicity assays.

### 2.2. Maintenance of Laboratorial Cultures of the Test Species

The species used in this study were selected based on several characteristics including their sensitivity to chemicals, easy maintenance under laboratory conditions, and well-known physiology, biology, and ecology. Furthermore, the species selected for this study have been extensively used in ecotoxicological studies as experimental models and are representative of different taxonomic and functional groups—[33,34,35,36]. Moreover, all media here used are standard, therefore, they are fully characterized or defined in the cited literature.

The cultures of the green unicellular microalgae *Raphidocelis subcapitata* (Chlorophyta, Sphaeropleales) were maintained in the laboratory in MBL medium [37] under axenic conditions and continuous light (intensity of 100 µE m/s) and room temperature of 23 ± 1 °C [34]. Culture renewal was performed once a week.

The cultures of *Daphnia magna* BEAK (Arthropoda, Cladocera) were maintained under controlled conditions of temperature (20 ± 2 °C) and photoperiod (16^L^:8^D^ h) in American Society for Testing and Materials hard water medium [38]. The culture medium was renewed every other day and organisms were fed daily with the green microalgae *R. subcapitata* (3 × 10^5^ cells/mL/day). The medium was also supplemented with the organic additive Marinure 25 (an extract from the algae *Ascophyllum nodosum*; Pann Britannica Industries Ltd., Waltham Abbey, UK). Organisms used for the ecotoxicity assays were newborns (less than 24 h old) released between the 3rd and 5th broods.

Regarding the freshwater rotifer *Brachionus calyciflorus* (Ploimida, Rotifera), neonates were obtained from cysts available in commercial kits (MicroBioTests, Ghent, Belgium). Hatching was performed at 23 °C for 24 h at a constant light intensity of 3000–4000 lux in ASTM moderately hard synthetic freshwater medium. Neonates were used to perform the ecotoxicity assays within 24 h after hatching (RoToxKit F ^®^ bench protocol).

The stock cultures of *Hydra viridissima* (Cnidaria, Anthoathecata) were maintained in the laboratory at room temperature of 20 ± 2 °C and 16^L^:8^D^ h photoperiod. The individuals of the stock culture were grown in reconstituted water following Trottier et al. [39] and fed with saltwater artemia (*Artemia salina*) hatched on the day. Feeding was performed twice a week for about one hour, after which the organisms were gently washed and transferred to fresh, clean medium.

The eggs of the *Danio rerio* fish were obtained after natural breeding of adult fish. The adults were maintained in a continuous water carbon filter flow-through system, where the physical and chemical parameters of the water were constantly monitored: temperature of 28 ± 1 °C, pH of 7.5 ± 0.5, electrical conductivity of 750 ± 50 µS/cm, and dissolved oxygen saturation level always above 95%. The fish were provided with a commercial artificial diet (ZM 400 Granular) twice a day. After mating, the eggs were collected and rinsed with water from the fish system and carefully selected under the stereomicroscope (Stereoscopic Zoom Microscope-SMZ 1500, Nikon Corporation). The screening of the eggs prior to the assay allowed to exclude non-fertilized eggs or embryos with deformities or wounds.

### 2.3. Lethal and Sublethal Assays

Information on the lethal and sublethal assays is described in detail in the sections below and summarized in Table S1 of the Supplementary Materials data file. The physicochemical parameters (pH, conductivity, and dissolved oxygen) were measured at two time points: at the beginning of the assay (with an extra portion of test solution made for this purpose) and at the end of the assay (due to small volumes of the assays carried out with algae, hydras, and fish, the volumes of all replicas were pooled to allow a sufficient high-water column for this purpose).

#### 2.3.1. Growth Assay with *R. subcapitata*

The assay performed with *R. subcapitata* lasted for 72 h. The OECD guideline 201 [33] was followed but adapted for 24-well plates after Moreira-Santos et al. [40]. Briefly, microalgae were exposed to a control consisting of MBL medium solely [37] and 7 concentrations of each salt described in Table S1. Three replicates were assembled for each treatment and each replicate consisted of 900 µL of test solution and 100 µL of an inoculum of *R. subcapitata* (at an initial cell density of 10^5^ cells/mL). Then, the test plates were incubated for 72 h in a climatic chamber at the temperature of 23 ± 1 °C and continuous white light (100 μE m/s). Absorbance (ABS) was used as a surrogate of cell density (D). At the beginning and end of the assay, algal growth was assessed by measuring the ABS at 440 nm through a Jenway 6505 UV/VIS spectrophotometer (Burlington, VT, USA). Then, the ABS obtained values were converted into cell density (D, cells/mL), followed by the determination of the average yield (Yield, cells/day) and specific growth rate (µ, day^−1^) for each tested treatment and control [33], as shown in the following Equations (1)–(3), respectively.

D (cell/mL) = −17,107.5 + (ABS × 7,925,350) (R^2^ = 0.99) (1)

(2) $$Yield_{i-j}=\frac{X_{j}-X_{i}}{t_{j}-t_{i},\;}\;(\mathrm{cells}/\mathrm{day}),$$

where *Yield_i−j_* is the average yield from time *i* to *j*; *X_i_* is the number of cells at time *i* (10^4^ cells); and *X_j_* is the number of cells at time *j* (2).

(3) $${µ}_{i-j}=\frac{lnX_{j}-lnX_{i}}{t_{j}-t_{i}}\;({\mathrm{day}}^{-1}),$$

where *µ_i_*_−*j*_ is the average specific growth rate from time *i* to *j*; *X_i_* is the biomass at time *i*; and *X_j_* is the cellular density at time *j* (3).

#### 2.3.2. The 48-h Mortality Assay with *D. magna*

The acute toxicity of each salt for *D. magna* neonates was evaluated following the standard procedure of the OECD guideline 202 [41]. Briefly, <24 h old neonates were exposed, for 48 h, to 5 concentrations of MgCl_2_ and CaCl_2_ and 6 concentrations of KCl (Table S1), plus a control (only ASTM). A total of four replicates were assembled per treatment, each with five neonates. Organisms are not fed during the exposure period, and the medium was not renewed. Assays were conducted at 20 ± 1 °C and with a light:dark cycle of 16^L^:8^D^. Assays were checked at 24 h and 48 h and organisms were considered dead when immobile for 15 s after gentle prodding.

#### 2.3.3. The 24-h Feeding Inhibition Assays with *D. magna*

Feeding inhibition was assessed by exposing the organisms to 7 concentrations of KCl, and 5 concentrations of CaCl_2_ or MgCl_2_ (Table S1). Prior to the beginning of the assays, newborns (from 3^rd^ brood) of *D. magna* were maintained in ASTM hardwater medium [38] and left to grow for 4 days. At this stage, groups of 5, 4-day-old neonates were randomly introduced in 50 mL glass vessels filled with 20 mL of test solution. Seven or five concentrations plus a control (ASTM hardwater) were tested (Table S1), with four replicates each. Organisms were fed for 24 h on the green algae *R. subcapitata* that was added in a concentration of 3.0 × 10^5^ cells/mL/day to each test vessel. Experiments were conducted at 20 ± 1 °C and in total darkness to avoid algal growth. Alongside, for each test, four blanks were used to guarantee that initial algal concentrations did not increase significantly during the exposure period. At the end of the 24 h exposure period, the organisms were removed and the remaining algae were resuspended. Absorbance (ABS) was measured at 440 nm (Jenway, 6505 UV/VIS spectrophotometer, Burlington, VT, USA) and converted to cell density (D, cell/mL) recurring to a previously established calibration curve for this specific green alga [3], as previously shown in Equation (1). The conversion into feeding rates (FR, cells/hour) followed Allen et al. [42], as shown in the following Equation (4).

(4) $$\mathrm{FR}=\frac{ln_{f}-ln_{i}}{t_{f}-t_{i}}\;(\mathrm{cells}/\mathrm{hour}),\;$$

where *ln_f_* is the final number of algae cells, *ln_i_* is the initial number of algae cells, and *t_f_* − *t_i_* (hours) is the time interval (4).

#### 2.3.4. Somatic Growth Rate Assay with *D. magna*

The 72-h somatic growth inhibition assay with *D. magna* was carried out using 8 concentrations in the case of CaCl_2_ and MgCl_2_, and 7 concentrations in the case of KCl (Table S1). Newborns (from 3^rd^ or 4^th^ broods), less than 24-h-old, were exposed individually in 50 mL glass vessels filled with 20 mL of test solution. As mentioned above, at least seven concentrations were tested plus a control (ASTM hardwater) (Table S1), each with ten replicates. The medium was supplied with algae (*R. subcapitata* at a concentration of 3.0 × 10^5^ cells/mL/day) and the organic extract. Organisms were fed daily, and the medium was renewed after 48 h of exposure. Growth rates were assessed by an organism’s measurement at 0 h and 72 h, using a stereomicroscope (Leica, MZ6). Values were then converted to daily juvenile growth (JGR, mm/day) following Burns CW [43] as follows in Equation (5):

(5) $$\mathrm{JGR}=\frac{ln_{f}-ln_{i}}{t_{f}-t_{i}}\;(\mathrm{mm}/\mathrm{day}),$$

where *l_f_* is the length of the organisms at the end of the assay (mm), *l_i_* is the initial length of the organisms (mm), and *t_f_* − *t_i_* (days) is the time interval (5).

#### 2.3.5. The 24-h Mortality and 48-h Reproduction Assays with *B. calyciflorus*

Both the 24-h mortality assay and the 48-h reproduction assay with the freshwater rotifer *B. calyciflorus* followed the RotoxKit F^®^ standard guideline (MicroBioTests, Ghent, Belgium). The 24-h mortality assay was assembled in 24-well plates and run at 23 ± 1 °C, in total darkness. Seven concentrations were assigned per each salt (Table S1) plus a control (consisting of medium only). A total of 5 replicates were performed per concentration and control, with five newly hatched (<24 h old) organisms per each replicate. No food was provided during the assay. The number of dead organisms was counted at the end of the 24 h of exposure. The 48 h reproduction assay was carried out under the same conditions of temperature and photoperiod, as described above for the acute exposure assay. The organisms were fed according to the standard guideline RotoxKit F^®^ chronic (MicroBioTests, Ghent, Belgium). At least five concentrations were assigned per salt and are described in Table S1 plus a control consisting of standard medium only. Eight replicates were established per treatment. At the beginning of the assay 1 single rotifer (<24 h old) was introduced in each replicate. After 48 h of incubation, the total number of swimming organisms per replicate was counted.

#### 2.3.6. The 96-h Mortality and Malformation Assay with *H. viridissima*

The effects of increased concentration of CaCl_2_, KCl, and MgCl_2_ were evaluated based on hydrozoan mortality rate and morphological malformations. The methodology for testing with hydra was based in Quinn et al. [36] and Trottier et al. [39]. A total of 7 concentrations were established for CaCl_2_ and 6 for MgCl_2_ and KCl (Table S1), plus a control (hydra medium solely), with 6 replicates per test treatment or control. Each replicate was filled with 2 mL solution and one hydranth. Test plates were incubated under the same conditions used for culturing [39]. Immediately at the beginning of the assay, organisms introduced in test plates were checked for signs of stress to assure that transference did not cause any injury. Then, every 24 h the organisms were checked for mortality and/or malformations [44]. The medium was not changed, and no food was added during the exposure period. The damage to the hydrozoans appeared in progressive stages, exhibiting morphological changes that were conventionally measured and expressed based on the observed changes in the animal. A score of 10 was attributed to healthy organisms according to Wilby [44]. When exposed to a toxic substance, hydras may start by showing signs of slight (clubbed tentacles) or more severe (shortened tentacles) intoxication. The next stage, the “tulip phase” will, in most cases, irreversibly lead to death of the organism (scoring below 6). The “post-tulip” phase ultimately leads to disintegration of the organism (score of 0; [39]).

#### 2.3.7. The 96-h Fish Embryo Acute Toxicity Assay (FET) with *Danio rerio*

The FET followed the OECD guideline 236 [35]. A total of 9 concentrations were established for CaCl_2_ and 8 for MgCl_2_ and KCl (Table S1), plus a control (consisting of only water from the zebrafish breeding system). Briefly, 10 embryos (healthy, without any visible malformation or abnormality, with ≤than 5 hpf (hours post-fertilization)) were randomly assigned individually per treatment and control in 24 well plates. Each well was previously filled with 2 mL of solution or only water from the system. During the 96 h of the assay, the medium was not renewed, and no food was added. Assays were run at 27 ± 1 °C and with a 16^L^:8^D^ photoperiod. Every 24 h the organisms were observed under a stereomicroscope (Stereoscopic Zoom Microscope—SMZ 1500, Nikon Corporation, Japan) and the following lethal and sublethal endpoints were reported: (a) survival; (b) malformations on the head, tail, and spine; (c) tail detachment; and (d) developmental stage [35].

## 3. Data Analysis

To facilitate the comparison of the results here obtained, the lethal and sublethal concentrations of each salt (MgCl_2_, KCl, and CaCl_2_) were converted to a common metric because of the different weights of the salts that can bias their apparent relative toxicity [6]. Moreover, since the main objective was to evaluate the toxicity driven by the cation and reasoned in previous studies [6], normalization was performed in relation to the overall molarity of the salt, but with emphasis on the cation molarity.

The lethal concentrations (expressed in relation to the chloride ion) causing 50% and 20% of the effect and respective confidence limits at 95% (LC_50_ and LC_20_, CL 95%) were calculated by fitting mortality data sets to Probit regression (PriProbit software; [45]). The effective concentrations (also expressed in relation to the ion chloride) causing 50% and 20% of effect and respective confidence limits at 95% (EC_50_ and EC_20_, CL 95%) were calculated by fitting the data sets to a three-parameter log-logistic model, using the software Sigma Plot 12.5 (Systat Software, Inc., San Jose, CA, USA). Significant differences between the LC_x_ and/or EC_x_ of salts within a species and/or endpoint were determined by direct comparison of the confidence limits at 95%, as this has been a recursive method in aquatic and soil ecotoxicology [46].

Finally, a literature search for median lethal and/or effective concentrations reported for NaCl, MgCl_2_, KCl, and CaCl_2_ and for the same species as the ones studied here or from the same ecological group was performed, aiming to provide an integrated overview of the suitability of each one of the studied salts as a surrogate of NaCl. In the present study, no ecotoxicological assays were carried out with NaCl, despite the comparison with this salt being a central role of the two specific objectives. This choice was based on the fact that a large dataset, specifically for the species studied here, is already available, thus avoiding further animal experiments (please see Venâncio et al. [23]).

## 4. Results

The toxicity of MgCl_2_, KCl, and CaCl_2_ was evaluated at lethal and sublethal levels in several freshwater species. The estimated LC_x,h_ and EC_x,h_ values are summarized in Table 1 and are presented in detail in the Supplementary Materials file (Figures S1–S8). The toxicity tests fulfilled the validity criteria specified in the respective guidelines and protocols [33,34,35,39], RoTox Kit F^®^ Acute, RoTox Kit F^®^ Chronic.

The physicochemical parameters (pH, conductivity, and dissolved oxygen) measured during the toxicity assays, warranting quality control criteria during the assays, are summarized in the Supplementary Materials, Table S2. The description and discussion of the results were made accounting for each cation molarity as the primary objective of this research was to make comparisons between cations. A surrogate measure (conductivity, mS/cm) of the tested concentrations at the beginning and end of the assays is provided in the Supplementary Materials, Table S2. Conductivity not only provides evidence of proper test solution preparation but also allows for comparison purposes regarding future work.

### 4.1. Lethal Assays

**Figure 1:**
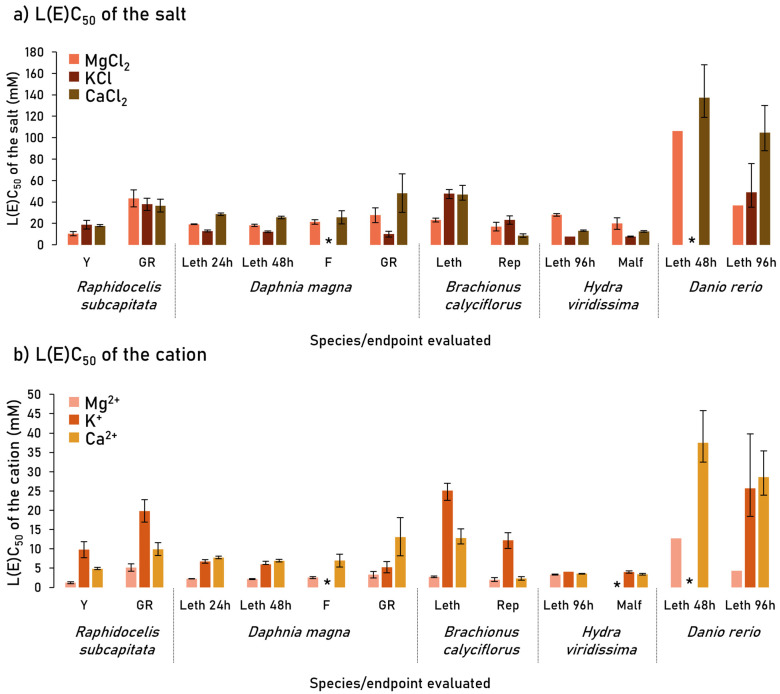
Median lethal and sublethal concentrations (mM) determined for (**a**) magnesium chloride (MgCl_2_), potassium chloride (KCl), and calcium chloride (CaCl_2_) salts and (**b**) the correspondent concentrations of each salt cation (Mg^2+^, K^+^, or Ca^2+^). Vertical bars correspond to the 95% confidence limits. * indicates that the parameter could not be estimated. Abbreviations stand for: Y—yield; GR—growth rate; Leth—lethality; F—feeding; Rep—reproduction; Malf—malformations.

As mentioned previously, to simplify comparisons between cations, the results were analyzed in relation to the molarity of each cation (Table 1; Figure 1; Supplementary Materials, Figures S1–S8). At the lethal level, overall Mg^2+^ was shown to be the most toxic cation of the three tested since its LC_50_s were significantly lower than the LC_50_s of the other cations in three out of four species: *D. magna* (48 h), *B. calyciflorus* (24 h), and *D. rerio* (96 h), for which the LC_50_s of 2.17, 2.77, and 3.35 mM Mg^2+^ were computed, respectively (Table 1; Figure 1). In *H. viridissima* all salts induced similar lethal toxicity. The K^+^ and Ca^2+^ induced lethal toxicity within the same range of concentrations with confidence limits overlapping in the cladoceran and the cnidarian (Table 1; Figure 1). Only in the case of the rotifer, Ca^2+^ was clearly more toxic than K^+^ with computed LC_50_s (CL 95%) of 12.8 (11.3–15.2) mM, and 25.1 (22.6–27.0) mM, respectively (Table 1; Figure 1). The cladoceran *D. magna* was consistently the most or one of the most sensitive organisms to lethal levels of all cations in a pairwise comparison with the rotifer in the case of K^+^ and with the cnidarian in Mg^2+^ and Ca^2+^ (Table 1; Figure 1). In contrast, the fish *D. rerio* was the most tolerant organism, and in the case of K^+^ and Ca^2+^ the difference between the fish and the most sensitive species was over three-fold (Table 1; Figure 1). The LC_20_ analysis (considered as a threshold for effect) showed trends similar to those described for the LC_50_s. Mg^2+^ was the most toxic at lethal levels in three out of four cases, followed by Ca^2+^ and K^+^ (Table 1). The primary consumers (daphnia and rotifer) and the cnidarian were the most sensitive species (Table 1). Considering the total composition of the salt (i.e., cation and anion), overall, KCl was the most toxic salt compared with MgCl_2_ and CaCl_2_, regardless of the median or threshold levels (LC_50_ or LC_20_; Table 1).

### 4.2. Sublethal Assays

Likewise to the lethal level, at the sublethal level and comparing the median concentrations, Mg^2+^ was the most toxic cation for *R. subcapitata*, and malformations were observed in *H. viridissima* (Table 1; Figure 1; Supplementary Materials, Figures S1–S8). In relation to *D. magna* feeding rates, Mg^2+^ was also the most toxic compared with Ca^2+^, although in this case for K^+^ no EC_x_ could be computed, whereas in the case of the rotifer *B. calyciflorus,* it shared with Ca^2+^ the highest toxicity (Table 1; Figure 1). The K^+^ was the least toxic for the alga and for the rotifer reproduction (Table 1; Figure 1). A higher diversity of responses was observed when comparing the species and endpoints at sublethal levels. The green alga *R. subcapitata* was both the most sensitive (yield) and the most tolerant (growth rate) to Mg^2+^ with EC_50_s of 1.24 and 5.17 mM, respectively. As for the cation K^+^, the hydra morphological state and the cladoceran growth rates were the most sensitive endpoints (with EC_50_s of 4.01- and 5.21-mM K^+^, respectively), while the green alga (growth rate) was the most tolerant (EC_50_ of 19.8 mM, Table 1). For Ca^2+^, the rotifer reproduction and the hydra morphology were the endpoints with the lowest EC_50_s (2.36 mM and 3.39 mM, respectively; Table 1; Figure 1). The cladoceran (growth rate) was the species that was most tolerant to Ca^2+^ (Table 1). At the EC_20_ level, Mg^2+^ was the most toxic in five out of a total of six cases, followed by Ca^2+^ and K^+^ as already observed at lethal levels (Table 1). Overall, similar to what was observed for lethal levels when considering total salt composition, at sublethal levels KCl was also the most toxic salt (EC_50_ or EC_20_; Table 1).

### 4.3. Comparing with Data for NaCl

To compare the ecotoxicity of the salts tested with that of NaCl, the median lethal or sublethal concentrations reported in the literature for the salts NaCl, MgCl_2_, KCl, and CaCl_2_ (expressed as cation molarity) were integrated with those obtained in the present study and are presented in the Supplementary Materials, Figure S9. This additional section aimed at acknowledging the paucity of comparative tests between NaCl and other salts (MgCl_2_, KCl, and CaCl_2_) since in many cases comparisons are not possible or highly uncertain due to lack of diversity, whether in terms of species or of endpoints (for example, rotifers and cladocerans). Comparing the data obtained from this study with those from previous reports by Venâncio et al. [14]—due to the proximity of the methodology and test media used—Na^+^ was not the most toxic cation. For instance, in the rotifer *B. calyciflorus* (mortality), the following crescent order of toxicity was observed: K^+^ < Na^+^ < Ca^2+^ < Mg^2+^, with Mg^2+^ being more than two-fold more toxic than Ca^2+^. Still, using the same example, whilst exposure to Na^+^ resulted in very similar values whether assessing mortality or reproduction (13.6- and 13.4-mM Na^+^, respectively), in the other three salts, the discrepancy between lethal and sublethal toxicity increased from 1.4- (Mg^2+^) to 5.4-fold (Ca^2+^). A similar pattern was observed for *D. magna* (mortality): Na^+^ induced the least toxicity among all four salts. Gonçalves et al. [47] reported an LC_50,48 h_ of 39.7 mM Na^+^, while in this study the values of 4.65 mM, 6.36 mM, and 6.96 mM were estimated for Mg^2+^, K^+^, and Ca^2+^, respectively. Although, when comparing the data obtained from this study with Mg^2+^ and Ca^2+^ with that obtained from Beisinger and Christensen [48], the effective concentrations obtained here were much higher than those previously reported. For instance, Beisinger and Christensen [48] reported an LC_50_ of 0.17 mM and 0.096 mM of Mg^2+^ and Ca^2+^, respectively, whereas we estimated LC_50,48 h_ values of 4.65 mM Mg^2+^ and 6.96 mM Ca^2+^. Others have stated NaCl as the least toxic salt compared with MgCl_2_ or CaCl_2_ for the cladoceran *D. magna* [18], contrary to what is reported here. It should be said that the organisms used in these studies are from hatching cysts (the result of sexual reproduction) and the tests were performed under different environmental conditions, namely, temperature, which could have induced differences in the results obtained [18].

## 5. Discussion

This study aimed to evaluate the toxicity of various salts with different cation compositions (MgCl_2_, KCl, and CaCl_2_) on several freshwater species. This provided novel, important, and updated data to the existing base literature, both for scenarios of climate change-driven salinization and/or human activity-induced salinization (road salt application). It also made possible the comparison of obtained data with the ecotoxicity of NaCl (a salt more frequently reported in the literature and often used to establish chloride (Cl^−^) safety values) and clarify the adequacy of using substitute salts for which safe values of salinities, more protective of the environment, could be defined.

Salt stress on freshwater species depends on the overall ionic composition of the salt, and not only on the overall chloride concentration. This happens because each ion is associated with a different toxicity degree or concentration [30]; thus, the overall toxicity may vary depending on the interaction and/or ratios between ions [6,49]. The cation may counteract the toxicity of the anion to some extent [6,49]. This hypothesis is partially supported by the MgCl_2_ salts studied here. For instance, when looking at the toxicity caused solely by the cation, this salt was the most toxic while when analyzing the total salt composition, this was not the case. On the other hand, association with a different cation may not always result in lower toxicity, and it becomes apparent that other cations, when in association with chloride, can be more toxic to some freshwater organisms than NaCl (as is the case with KCl—also in the present study with lower toxicity values when looking solely to the cation comparatively to the salt [50,51,52,53,54,55,56,57,58]). For instance, Davis et al. [51] verified that adult zebra mussels (*Dreissena polymorpha*) died within 24 h after being exposed to a NaCl level of 30 g/L, while when exposed to 30 g/L of KCl, death occurred within 6 h of exposure [51]. Likewise, Lombardi et al. [52] reached a similar conclusion when assessing the toxicity of NaCl and KCl to the microcrustacean *Ceriodaphnia dubia*, with reported median chronic toxicity values (EC_50,7 days_) of 0.2 g KCl/L and 0.5 g NaCl/L, respectively, representing an over two-fold difference between the two salts [52]. Moreover, Szklarek et al. [18] reported for three filter-feeder species (*C. dubia*, *B. calyciflorus*, and *Thamnocephalus platyurus*), that NaCl induced the least toxicity compared with CaCl_2_ or MgCl_2_ [18]. Thus, from what is presented above it seems to be fundamental that the derivation of updated data must be based on ion-specific toxicity, but also that the integration of different ecological groups and/or species along with other endpoints besides mortality must follow up as fundamental points.

Overall, the ranking of cation toxicity in this study was: K^+^ > Na^+^ > Mg^2+^ ≈ Ca^2+^. In Figure S9 of the Supplementary Materials data file it can be noticed that the K^+^ values appear clustered at lower values than those of the other three salts. Although all ionic forms discussed here perform important cellular functions and contribute to the overall homeostatic state of organisms, the highest toxicity of K^+^ may be related to its role as a major component of intracellular fluids. As it is responsible for cellular signal transduction, K^+^ is maintained at greater concentrations inside the cell comparatively to extracellular fluids (its uptake is an energy expenditure process as it occurs against the gradient), while the opposite is valid for Na^+^ (it is maintained mostly outside the cell) [29,53]. Thus, one might hypothesize that such a high K^+^ concentration inside the cell is in very fine balance and might be easily turned over when organisms are confronted with increasing exterior concentrations, deregulating the external uptake of Na^+^ that is dependent and regulated by the intracellular K^+^ gradient, leading to ionic and cell volume imbalances [29]. Regarding the two other salts, the toxicity of Mg^2+^ was, in most cases, very similar to that of Ca^2+^. Ca^2+^ transport occurs at the expense of energy; thus, osmoregulation might be a very expensive metabolic process. Ca^2+^ is present at very low intracellular levels, and even inside the cell it remains largely associated with protein and phospholipids because when free it easily induces intracellular toxic effects [29]. Despite its role as a secondary messenger, it performs countless functions in the cell depending on it [53,54]. Notwithstanding, its lower toxicity compared with the other studied cations might be related to two processes: first, its transport is conditioned immediately before entering the cell because the entrance of Ca^2+^ is mediated by an exchanger that also transports Na^+^, and thus outside the cell both ions compete for the same binding site; as well, its transport might also be inhibited by other divalent ions (such as Zn^2+^ or Cd^2+^; [29]); secondly, organisms possess very efficient mechanisms for Ca^2+^ removal and excretion from the cell either in the dissolved form or in complexes that can be stored in bones, shells, or exoskeletons [55]. Mg^2+^ lower toxicity is possibly related to its performance in cells sugar metabolism as well as in nucleic acids degradation since the cleavage of phosphate bonds are magnesium mediated [53]. Moreover, Mg^2+^ is a limiting nutrient for plants, and its deficiency results in lower carbon dioxide fixation and plant biomass/growth [56,57]. However, it must also be mentioned that the proximity between the toxicity levels of both ions may be explained by Mg^2+^ being considered an antagonist (blocker) of Ca^2+^, thus competing in the cells for the same binding sites [26]. Such a competing nature between both ions has been previously discussed in works focused on the medium hardness, which in part may corroborate why Mg^2+^ showed toxicity similar to that of Ca^2+^ in cladocerans. In part, Ca^2+^ plays a fundamental role in the life cycle traits of these organisms, but also it must be considered that Mg^2+^ might have impeded the uptake of Ca^2+^ from the medium due to ionic antagonism, the reason for which, in some cases, Mg^2+^ presented lower toxicity [26].

The lack of data for a large number of species and/or trophic guilds (cnidarians, rotifers), as well as for endpoints (most of the results report to lethal data), is one of the main obstacles in the construction of relevant and integrated risk assessment frameworks on salinization of freshwater ecosystems, which primarily depend on the delivery of extensive and relevant data. Much of the information available lacks important ecological groups, and detailed information on the methodology employed is not always is presented, possibly leading to inadequate risk estimations. The example of the NaCl salt from which the acute chloride criterion (USEPA) was derived was based (only) on the ecotoxicity it poses to three species (one filter-feeding microcrustacean and two fish). This guideline has been in force since 1988 and was derived from a chronic criterion for Cl^−^ based on acute toxicity data because there were not enough data available. For chloride, the much-desired establishment (or update) of a guideline on cations should not fall under such deviation. The values derived here are, therefore, important for cementing recent databases that foster the inclusion of other salts that may pose a hazard to freshwater ecosystems [18,31,58]. In addition, when comparisons were possible, it was evident that, in many cases, the values were much different. The authors must appeal to the report whenever possible for media composition and/or the use of standard synthetic media fully characterized by international guidelines, and test conditions must always be clearly stated. This is of utmost importance because differences in media composition may result in different outcomes that overshadow comparison [59,60]. For instance, the discrepancy between these results and those obtained by Beisinger and Christensen [48] might be driven by water hardness, since the authors used lake water and here synthetic hard water was used. Previous studies concluded that water hardness reduces toxicity [6,60].

The results provided here may serve as support points for: (i) databases directly linked to saline intrusion due to sea level rise, and (ii) databases related to the use of road deicing salts. In relation to the first point, it stands out that salts other than NaCl may have toxic effects that can be incorporated into more conservative frameworks (to be understood as a worst-case scenario; [52]). Regarding the second point, the results obtained here suggest that the use of other salts in deicing operations (MgCl_2_ or CaCl_2_) might be a safer alternative to NaCl, as they might induce less toxicity (freshwater biota near road tracks). Despite other studies pointing in a different direction, a broader number of species and endpoints were evaluated here [18]. Moreover, these surrogate salts, MgCl_2_ or CaCl_2_, aside from being suggested as more environmentally friendly, might be more effective leading to the possible application of lower quantities (and lower associated costs) because of their specific eutectic temperatures [16]. The salt NaCl has an eutectic point of −6 °F, while those of MgCl_2_ and CaCl_2_ are −28 °F and −60 °F, respectively. This indicates that the latter can act and be effective over a wider range of temperatures [16]. The advance of seawater and road deicing operations cannot be stopped. However, the enrichment of databases, the sharing of information to regulatory agents, and the proposal of new and better alternatives to be employed, aiming at more sustainable human and environmental frameworks, should always be a reason for research in the area of salinization.

## 6. Conclusions

The establishment of specific ion toxicity-oriented frameworks for the protection of freshwater ecosystems has been desired for a long time. The present work provided ecotoxicological effects for three major ions (K^+^, Mg^2+^, and Ca^2+^) and for hitherto little studied ecological groups. *Hydra viridissima* and the primary consumers, *D. magna* and *B. calyciflorus*, were the most sensitive organisms to all studied ions, proving to be suitable bioindicators of poor-quality freshwaters. A further evaluation of the data here provided with data reported in the literature for a very common surrogate salt (NaCl), showed that: (i) K^+^ was more toxic than Na^+^, and thus could be proposed as a more conservative surrogate with respect to lower tiers of the risk assessment of salinization due to sea level rise; and (ii) Ca^2+^ was the least toxic cation of all tested suggesting that the employment of calcium-based deicing salts might be a safer and eco-friendlier alternative to the others. The information provided here contributes to reduce the salinization problems associated with the use of deicer salts, as well as assisting in the definition of prevention strategies that might minimize the entrance of seawater up to levels that can be supported by freshwater ecosystems. Thus, it can contribute substantially to providing up-to-date information for the purpose of integrating more comprehensive and realistic framework structures that can be presented to regulators.

## Figures and Tables

**Table 1:** Summary of the concentrations (expressed in molarity) causing 50% and 20% of effect after *h* hours of exposure [L(E)C*_x_*_,*h*_] to increased concentrations of magnesium chloride (MgCl_2_), potassium chloride (KCl), and calcium chloride (CaCl_2_) salts and respective correspondence in relation to the respective cation concentration (Mg^2+^, K^+^, or Ca^2+^). Confidence limits at 95% (CL 95%) are depicted inside parenthesis.

Species	Endpoint	L(E)C*_x_*_,*h*_ (CL 95%)	MgCl_2_·6H_2_O mM	Mg^2+^ mM	KCl mM	K^+^ mM	CaCl_2_·2H_2_O mM	Ca^2+^ mM
*Raphidocelis subcapitata*	Yield	EC_50,72h_	10.3 (8.36–12.3)	1.24 **A** (1.00–1.47)	18.8 (14.8–22.8)	9.85 **B** (7.74–11.9)	17.7 (17.0–19.0)	4.82 **C** (4.64–5.19)
		EC_20,72h_	x>	x>	6.71 (4.02–8.05)	3.52 **A** (2.11–4.22)	12.9 (11.6–14.3)	3.52 **A** (3.15–3.89)
	Growth rate	EC_50,72h_	43.2 (35.5–51.2)	5.17 **A** (4.25–6.12)	37.8 (32.2–43.5)	19.8 **B** (16.9–22.8)	36.5 (30.5–42.5)	9.94 **C** (8.33–11.6)
		EC_20,72h_	13.4 (8.76–18.0)	1.60 **A** (1.05–2.15)	15.2 (11.0–19.5)	7.95 **B** (5.77–10.2)	20.0 (14.2–25.7)	5.45 **B** (3.88–7.01)
*Daphnia magna*	Mortality	LC_50,24h_	19.2 (18.9–19.5)	2.29 **A** (2.26–2.33)	12.9 (11.9–13.7)	6.75 **B** (6.26–7.17)	28.5 (27.3–29.7)	7.77 **B** (7.44–8.10)
		LC_20,24h_	18.7 (18.4–19.0)	2.24 **A** (2.21–2.28)	11.0 (9.79–11.7)	5.77 **B** (5.13–6.12)	26.7 (24.9–27.7)	7.27 **B** (6.79–7.57)
	Mortality	LC_50,48h_	18.2 (17.1–19.2)	2.17 **A** (2.05–2.30)	12.2 (11.4–13.0)	6.40 **B** (5.98–6.82)	25.5 (24.4–26.7)	6.95 **B** (6.64–7.29)
		LC_20,48h_	16.0 (14.7–16.9)	1.91 **A** (1.75–2.02)	10.6 (9.39–11.3)	5.56 **B** (4.92–5.91)	23.6 (21.8–24.7)	6.43 **B** (5.95–6.73)
	Feeding	EC_50,24h_	21.4 (19.2–23.5)	2.55 **A** (2.30–2.81)	x>	x>	25.6 (19.5–31.8)	6.99 **B** (5.30–8.66)
		EC_20,24h_	20.0 (17.2–22.8)	2.39 **A** (2.06–2.72)	x>	x>	16.6 (9.05–26.3)	4.52 **A** (2.47–7.16)
	Growth	EC_50,72h_	27.6 (20.8–34.5)	3.30 **A** (2.48–4.12)	9.93 (7.24–12.7)	5.21 **AB** (3.80–6.68)	48.2 (30.2–66.3)	13.1 **B** (8.23–18.1)
		EC_20,72h_	13.0 (10.9–15.2)	1.56 **A** (1.30–1.81)	3.76 (1.61–5.90)	1.97 **AB** (0.84–3.09)	14.4 (9.73–19.2)	3.93 **B** (2.65–5.23)
*Brachionus calyciflorus*	Mortality	LC_50,24h_	23.2 (21.4–25.0)	2.77 **A** (2.55–2.98)	47.9 (43.1–51.5)	25.1 **B** (22.6–27.0)	47.0 (41.6–55.6)	12.8 **C** (11.3–15.2)
		LC_20,24h_	17.8 (15.5–19.6)	2.13 **A** (1.86–2.34)	38.6 (31.5–42.9)	20.3 **B** (16.5–22.5)	28.4 (22.3–32.6)	7.73 C (6.08–8.90)
	Reproduction	EC_50,48h_	17.0 (12.9–21.1)	2.03 **A** (1.54–2.52)	23.2 (19.2–27.1)	12.2 **B** (10.1–14.2)	8.64 (7.01–10.3)	2.36 **A** (1.91–2.80)
		EC_20,48h_	11.1 (6.10–16.2)	1.33 **A** (0.73–1.94)	11.9 (6.44–17.4)	6.26 **B** (3.38–9.15)	8.30 (5.85–10.8)	2.26 **A** (1.59–2.93)
*Hydra viridissima*	Mortality	LC_50,48h_	x>	x>	8.32 (7.38–9.39)	4.36 **A** (3.87–4.92)	18.5 (18.0–19.0)	5.04 **A** (4.91–5.17)
		LC_20,48h_	27.2 (26.0–28.3)	3.25 **A** (3.11–3.39)	7.78 (5.23–8.32)	4.08 **A** (2.74–4.36)	18.1 (17.6–18.6)	4.93 **B** (4.80–5.06)
	Mortality	LC_50,96h_	28.0 (26.8–29.2)	3.35 **A** (3.21–3.49)	7.78 (-)	4.08 **A** (-)	13.1 (12.7–13.3)	3.58 **A** (3.47–3.62)
		LC_20,96h_	27.2 (-)	3.25 **A** (-)	6.71 (-)	3.52 **A** (-)	12.9 (12.5–13.3)	3.50 **A** (3.39–3.62)
	Malformation	EC_50,48h_	x>	x>	7.91 (7.78–8.18)	4.15 **A** (4.08–4.29)	17.0 (16.1–17.8)	4.64 **A** (4.38–4.86)
		EC_20,48h_	6.25 (-)	0.75 **A** (-)	7.51 (7.11–7.78)	3.94 **B** (3.73–4.08)	15.6 (14.3–17.1)	4.26 **B** (3.89–4.65)
	Malformation	EC_50,96h_	19.8 (14.5–25.1)	2.37 **A** (1.74–3.00)	7.65 (7.11–8.18)	4.01 **B** (3.73–4.29)	12.5 (11.8–13.2)	3.39 **B** (3.21–3.60)
		EC_20,96h_	7.97 (3.10–12.8)	0.95 **A** (0.37–1.53)	6.98 (5.63–8.32)	3.66 **B** (2.95–4.36)	11.0 (9.80–12.0)	2.99 **B** (2.67–3.28)
*Danio rerio*	Mortality	LC_50,48h_	106.2 (-)	12.7 **A** (-)	x>	x>	137.4 (119.0–168.0)	37.5 **B** (32.5–45.8)
		LC_20,48h_	67.4 (-)	8.06 **A** (-)	x>	x>	71.4 (54.5–82.3)	19.5 **B** (14.9–22.4)
	Mortality	LC_50,96h_	36.7 (-)	4.35 **A** (-)	49.1 (35.0–75.9)	25.7 **B** (18.4–39.8)	104.8 (87.8–129.9)	28.6 **B** (23.9–35.4)
		LC_20,96h_	31.4 (24.2–35.2)	3.76 **A** (2.89–4.21)	18.4 (8.58–23.7)	9.64 **B** (4.50–12.5)	48.6 (36.5–57.8)	13.2 **AB** (9.96–15.7)

x>—greater than the highest tested concentration. Capital letters (**A**, **B**, **C**) are used to indicate homogenous groups, within the same species and endpoint, of salts in which an overlap of the confidence limits (95%) of L(E)C_50_ or L(E)C_20_ occurred [46].

## Data Availability

Data available upon request.

## Supplementary Materials

The following supporting information can be downloaded at: https://www.mdpi.com/article/10.3390/ijerph20031741/s1, Table S1: Summary of the procedures used to perform the ecotoxicity assays with each tested species, including the range of concentrations tested for each salt. CTR stands for Control; Figure S1: Yield (number of cells; graphics a to c) and average growth rate (µ.day^−1^; graphics d to e) of the green unicellular algae *Raphidocelis subcapitata* after 72 h of exposure to increased concentrations of three salts (magnesium chloride—MgCl_2_; potassium chloride—KCl; and calcium chloride—CaCl_2_); Figure S2: Survival percentage of *Daphnia magna* after 24 h (black bars) and 48 h (gray bars) of exposure to increased concentrations of three salts (magnesium chloride—MgCl_2_; potassium chloride—KCl; and calcium chloride—CaCl_2_); Figure S3: Feeding rates (algae cells ingested/hour) of *Daphnia magna* after 24 h of exposure to increased concentrations of three salts (magnesium chloride—MgCl2; potassium chloride—KCl; and calcium chloride—CaCl_2_); Figure S4: Daily growth rate (µ; mm.day^−1^) of *Daphnia magna* after 72 h of exposure to increased concentrations of three salts (magnesium chloride—MgCl_2_; potassium chloride—KCl; and calcium chloride—CaCl_2_); Figure S5: Survival percentage (a trough c) and total reproduction (as the number of released offspring; d trough f) of the freshwater rotifer *Brachionus calyciflorus* after 24 h and 48 h of exposure, respectively, to increased concentrations of three salts (magnesium chloride—MgCl_2_; potassium chloride—KCl; and calcium chloride—CaCl_2_); Figure S6: Survival percentage of the cnidarian *Hydra viridissima* after 48 h and 96 h of exposure (dark and light gray bars, respectively), to increased concentrations of three salts (magnesium chloride—MgCl_2_; potassium chloride—KCl; and calcium chloride—CaCl_2_); Figure S7: Malformation scoring (according to Wilby, 1988) attributed to the cnidarian *Hydra viridissima* after 48 h and 96 h of exposure (pattern bars and full bars, respectively), to increased concentrations of three salts (magnesium chloride—MgCl_2_; potassium chloride—KCl; and calcium chloride—CaCl_2_); Figure S8: Survival percentage of *Danio rerio* embryos exposed for 48 h (black bars) and 96 h (gray bars) to increased concentrations of three salts (magnesium chloride—MgCl_2_; potassium chloride—KCl; and calcium chloride—CaCl_2_). Concentrations are expressed in chloride (Cl^−^; g/L); Figure S9: Comparison of the median lethal and sublethal concentrations of the salts sodium chloride (NaCl), magnesium chloride (MgCl_2_), potassium chloride (KCl), and calcium chloride (CaCl_2_), expressed in molarity of the respective cation, obtained in this study and collected from previous studies; Table S2: Range of values for the parameters measured at the beginning and end of each assay (highest and lowest pH, conductivity, and dissolved oxygen) for each of the three tested salts (magnesium chloride—MgCl_2_, calcium chloride—CaCl_2_, and potassium chloride—KCl). References [61,62] are cited in the supplementary materials.

## References

[1] Leitao J., Ribeiro R., Soares A.M., Lopes I. (2013). Tolerance to Copper and to Salinity in *Daphnia longispina*: Implications within a Climate Change Scenario. PLoS ONE.

[2] Coldsnow K.D., Mattes B.M., Hintz W.D., Relyea R.A. (2017). Rapid evolution of tolerance to road salt in zooplankton. Environ. Pollut..

[3] Venâncio C., Anselmo E., Soares A., Lopes I. (2017). Does increased salinity influence the competitive outcome of two producer species?. Environ. Sci. Pollut. Res..

[4] Schulz C.-J., Cañedo-Argüelles M. (2019). Lost in translation: The German literature on freshwater salinization. Philos. Trans. R. Soc. B Biol. Sci..

[5] Jackson J.K., Funk D.H. (2018). Temperature affects acute mayfly responses to elevated salinity: Implications for toxicity of road de-icing salts. Philos. Trans. R. Soc. B.

[6] Mount D.R., Gulley D.D., Hockett J.R., Garrison T.D., Evans J.M. (1997). Statistical models to predict the toxicity of major ions to *Ceriodaphnia dubia*, *Daphnia magna* and *Pimephales promelas* (fathead minnows). Environ. Toxicol. Chem. Int. J..

[7] Leite T., Santos J.M., Ferreira M.T., Canhoto C., Branco P. (2019). Does short-term salinization of freshwater alter the behaviour of the Iberian barbel (*Luciobarbus bocagei*, Steindachner 1864)?. Sci. Total Environ..

[8] Venâncio C., Ribeiro R., Lopes I. (2020). Active emigration from climate change-caused seawater intrusion into freshwater habitats. Environ. Pollut..

[9] Gutierrez M.F., Tavşanoğlu N., Vidal N., Yu J., Mello F.T.-D., Çakiroglu A.I., He H., Liu Z., Jeppesen E. (2018). Salinity shapes zooplankton communities and functional diversity and has complex effects on size structure in lakes. Hydrobiologia.

[10] Gutiérrez-Cánovas C., Sánchez-Fernández D., Cañedo-Argüelles M., Millán A., Velasco J., Acosta R., Fortuno P., Otero N., Soler A., Bonada N. (2019). Do all roads lead to Rome? Exploring community trajectories in response to anthropogenic salinization and dilution of rivers. Philos. Trans. R. Soc. B.

[11] Wang N., Kunz J.L., Dorman R.A., Ingersoll C.G., Steevens J.A., Hammer E.J., Bauer C.R. (2018). Evaluation of chronic toxicity of sodium chloride or potassium chloride to a unionid mussel (*Lampsilis siliquoidea*) in water exposures using standard and refined toxicity testing methods. Environ. Toxicol. Chem..

[12] Wang N., Ivey C.D., Dorman R.A., Ingersoll C.G., Steevens J., Hammer E.J., Bauer C.R., Mount D.R. (2018). Acute toxicity of sodium chloride and potassium chloride to a unionid mussel (*Lampsilis siliquoidea*) in water exposures. Environ. Toxicol. Chem..

[13] Ofoegbu P.U., Campos D., Soares A.M.V.M., Pestana J.L.T. (2019). Combined effects of NaCl and fluoxetine on the freshwater planarian, *Schmidtea mediterranea* (Platyhelminthes: Dugesiidae). Environ. Sci. Pollut. Res..

[14] Venâncio C., Castro B.B., Ribeiro R., Antunes S., Abrantes N., Soares A., Lopes I. (2019). Sensitivity of freshwater species under single and multigenerational exposure to seawater intrusion. Philos. Trans. R. Soc. B Biol. Sci..

[15] Venâncio C., Castro B., Ribeiro R., Antunes S., Lopes I. (2019). Sensitivity to salinization and acclimation potential of amphibian (*Pelophylax perezi*) and fish (*Lepomis gibbosus*) models. Ecotoxicol. Environ. Saf..

[16] Kelting D.L., Laxson C.L. (2010). Review of Effects and Costs of Road De-Icing with Recommendations for Winter Road Management in the Adirondack Park.

[17] Ružinskas A., Bulevičius M., Sivilevičius H. (2016). Laboratory investigation and efficiency of deicing materials used in road maintenance. Transport.

[18] Szklarek S., Górecka A., Salabert B., Wojtal-Frankiewicz A. (2022). Acute toxicity of seven de-icing salts on four zooplankton species–is there an “eco-friendly” alternative?. Ecohydrol. Hydrobiol..

[19] Valleau R.E., Celis-Salgado M.P., Arnott S.E., Paterson A.M., Smol J.P. (2022). Assessing the Effect of Salinization (NaCl) on the Survival and Reproduction of Two Ubiquitous Cladocera Species (*Bosmina longirostris* and *Chydorus brevilabris*). Water Air Soil Pollut..

[20] Coldsnow K.D., Relyea R.A. (2018). Toxicity of various road-deicing salts to Asian clams (*Corbicula fluminea*). Environ. Toxicol. Chem..

[21] Hintz W.D., Relyea R.A. (2017). Impacts of road deicing salts on the early-life growth and development of a stream salmonid: Salt type matters. Environ. Pollut..

[22] Evans M., Frick C. (2001). The Effects of Road Salts on Aquatic Ecosystems.

[23] Venâncio C., Ribeiro R., Lopes I. (2021). Seawater intrusion: An appraisal of taxa at most risk and safe salinity levels. Biol. Rev..

[24] Cañedo-Argüelles M., Kefford B.J., Piscart C., Prat N., Schäfer R.B., Schulz C.-J. (2013). Salinisation of rivers: An urgent ecological issue. Environ. Pollut..

[25] IPPC. https://www.ipcc.ch/site/assets/uploads/sites/3/2019/11/09_SROCC_Ch05_FINAL-1.pdf.

[26] Van Dam R.A., Hogan A.C., McCullough C.D., Houston M.A., Humphrey C.L., Harford A.J. (2010). Aquatic toxicity of magnesium sulfate, and the influence of calcium, in very low ionic concentration water. Environ. Toxicol. Chem..

[27] Pérez-Fuentetaja A., Goodberry F. (2016). Daphnia’s challenge: Survival and reproduction when calcium and food are limiting. J. Plankton Res..

[28] Shetty P., Gitau M.M., Maróti G. (2019). Salinity Stress Responses and Adaptation Mechanisms in Eukaryotic Green Microalgae. Cells.

[29] Griffith M.B. (2016). Toxicological perspective on the osmoregulation and ionoregulation physiology of major ions by freshwater animals: Teleost fish, crustacea, aquatic insects, and Mollusca. Environ. Toxicol. Chem..

[30] Kunz J.L., Conley J.M., Buchwalter D.B., Norberg-King T.J., Kemble N.E., Wang N., Ingersoll C.G. (2013). Use of reconstituted waters to evaluate effects of elevated major ions associated with mountaintop coal mining on freshwater invertebrates. Environ. Toxicol. Chem..

[31] Bogart S.J., Azizishirazi A., Pyle G.G. (2018). Challenges and future prospects for developing Ca and Mg water quality guidelines: A meta-analysis. Philos. Trans. R. Soc. B.

[32] Soucek D.J., Mount D.R., Dickinson A., Hockett J.R. (2018). Influence of dilution water ionic composition on acute major ion toxicity to the mayfly *Neocloeon triangulifer*. Environ. Toxicol. Chem..

[33] OECD (2004). Test No. 201: Freshwater Alga and Cyanobacteria, Growth Inhibition Test.

[34] OECD (2006). Lemna sp. Growth Inhibition Test. Test Guideline 221. Guidelines for Testing of Chemicals.

[35] OECD (2013). OECD Guidelines for the Testing of Chemicals. Test Guideline 236. Fish Embryo Acute Toxicity (FET) Test.

[36] Quinn B., Gagné, F, Blaise, C (2012). Hydra, a model system for environmental studies. Int. J. Dev. Biol..

[37] Nichols H.W., Stein J.R. (1973). Handbook of Phycological Methods.

[38] ASTM—American Society of Testing and Materials (2002). Standard Guide for Conducting Acute Toxicity Tests on Test Materials with Fishes, Microinvertebrates, and Amphibians. Annual Book of ASTM Standards. 1105.

[39] Trottier S., Blaise C., Kusui T., Johnson E.M. (1997). Acute toxicity assessment of aqueous samples using a microplate-based Hydra attenuata assay. Environ. Toxicol. Water Qual. Int. J..

[40] Moreira-Santos M., Soares A.M., Ribeiro R. (2004). An in situ bioassay for freshwater environments with the microalga *Pseudokirchneriella subcapitata*. Ecotoxicol. Environ. Saf..

[41] OCDE (2004). Daphnia sp., Acute Immobilisation Test. Test Guideline 202. Guidelines for Testing of Chemicals.

[42] Allen Y., Calow P., Baird D.J. (1995). A mechanistic model of contaminant-induced feeding inhibition in *Daphnia magna*. Environ. Toxicol. Chem. Int. J..

[43] Burns C.W. (2000). Crowding-Induced changes in growth, reproduction and morphology of *Daphnia*. Freshw. Biol..

[44] Wilby O.K. (1988). The Hydra regeneration assay. Proceedings of Workshop Organised by Association Francaise de Teratologie.

[45] Sakuma M. (1998). Probit analysis of preference data. Appl. Entomol. Zool..

[46] Azimonti (2016). Comparison of NOEC Values to EC10/EC20 Values, Including Confidence Intervals, in Aquatic and Terrestrial Ecotoxicological Risk Assessment.

[47] Gonçalves A.M., Castro B.B., Pardal M.A., Gonçalves F. (2007). Salinity effects on survival and life history of two freshwater cladocerans (*Daphnia magna* and *Daphnia longispina*). Ann. Limnol. Int. J. Limnol..

[48] Beisinger K.E., Christensen G.M. (1972). Effects of various metals on survival, growth, reproduction, and metabolism of *Daphnia magna*. J. Fish. Board Can..

[49] Elphick J.R.F., Bergh K.D., Bailey H.C. (2011). Chronic toxicity of chloride to freshwater species: Effects of hardness and implications for water quality guidelines. Environ. Toxicol. Chem..

[50] Ivey C.D., Besser J.M., Ingersoll C.G., Wang N., Rogers D.C., Raimondo S., Bauer C.R., Hammer E.J. (2016). Acute sensitivity of the vernal pool fairy shrimp, *Branchinecta lynchi* (Anostraca; Branchinectidae), and surrogate species to 10 chemicals. Environ. Toxicol. Chem..

[51] Davis E.A., Wong W.H., Harman W.N. (2018). Toxicity of potassium chloride compared to sodium chloride for zebra mussel decontamination. J. Aquat. Anim. Health.

[52] Lombardi J.V., Bazante-Yamaguishi R., Madeira F.F., Concilio P.L., Caruso N.P.P., Barbieri E. (2018). Potassium chloride and sodium chloride as reference toxicants to assess quality of toxicity tests carried out with the microcrustacean cladocera *Ceriodaphnia dubia*. Bol. do Inst. de Pesca.

[53] Matsarskaia O., Roosen-Runge F., Schreiber F. (2020). Multivalent ions and biomolecules: Attempting a comprehensive perspective. ChemPhysChem.

[54] Sakai T., Yamamoto T., Matsubara S., Kawada T., Satake H. (2020). Invertebrate Gonadotropin-Releasing Hormone Receptor Signaling and Its Relevant Biological Actions. Int. J. Mol. Sci..

[55] Alstad N.E.W., Skardal L., Hessen D.O. (1999). The effect of calcium concentration on the calcification of *Daphnia magna*. Limnol. Oceanogr..

[56] Hauer-Jákli M., Tränkner M. (2019). Critical Leaf Magnesium Thresholds and the Impact of Magnesium on Plant Growth and Photo-Oxidative Defense: A Systematic Review and Meta-Analysis From 70 Years of Research. Front. Plant Sci..

[57] Soundararajan P., Manivannan A., Ko C.H., Park J.E., Jeong B.R. (2019). Evaluation of relative toxicity caused by deicing agents on photosynthesis, redox homeostasis, and the osmoregulatory system in creeper-type plants. Hortic. Environ. Biotechnol..

[58] Schuler M.S., Cañedo-Argüelles M., Hintz W.D., Dyack B., Birk S., Relyea R.A. (2018). Regulations are needed to protect freshwater ecosystems from salinization. Philos. Trans. R. Soc. B.

[59] Soucek D.J., Linton T.K., Tarr C.D., Dickinson A., Wickramanayake N., Delos C.G., Cruz L.A. (2011). Influence of water hardness and sulfate on the acute toxicity of chloride to sensitive freshwater invertebrates. Environ. Toxicol. Chem..

[60] Mount D.R., Erickson R.J., Highland T.L., Hockett J.R., Hoff D.J., Jenson C.T., Norberg-King T.J., Peterson K.N., Polaske Z.M., Wisniewski S. (2016). The acute toxicity of major ion salts to *Ceriodaphnia dubia*: I. Influence of background water chemistry. Environ. Toxicol. Chem..

[61] Freiry R., Stelzer J.A.A., Maltchik L., Arenzon A. (2014). Sensitivity of *Danio rerio* (Teleostei, Cyprinidae) during two stages of development based on acute toxicity tests. Bull. Environ. Contam. Toxicol..

[62] Simmons J.A. (2012). Toxicity of major cations and anions (Na^+^, K^+^, Ca^2+^, Cl^−^, and SO) to a macrophyte and an alga. Environ. Toxicol. Chem..

