# Supplementary material for: Cation Composition Influences the Toxicity of Salinity to Freshwater Biota
Source: Int J Environ Res Public Health. 2023 Jan 18;20(3):1741. doi: 10.3390/ijerph20031741 (PMC9914514; doi:10.3390/ijerph20031741)
Supplement: Supplementary file 1 [file ijerph-20-01741-s001.zip › ijerph-2158727-supplementary.pdf]

International Journal of
Environmental Research and Public Health

MDPI

Article

Cation Composition Influences the Toxicity of Salinity to Freshwater Biota

Cátia Venâncio *, Karen Caon and Isabel Lopes *

Centre for Marine and Environmental Studies (CESAM) and Department of Biology, University of Aveiro, Campus Universitário de Santiago, 3810-193 Aveiro, Portugal
* Correspondence: a32884@ua.pt (C.V.); ilopes@ua.pt (I.L.)

Supplementary data

Table S1: Summary of the procedures used to perform the ecotoxicity assays with each tested species, including the range of concentrations tested for each salt. CTR stands for Control.

Species	Endpoint<br>Assay duration (hours)	Guideline/Protocol	Dilution medium<br>Dilution factor	Nº replicates<br>Org. per replicate	[KCl]<br>g/L	[$CaCl_2$]<br>g/L	[$MgCl_2$]<br>g/L
Raphidocelis subcapitata	Yield<br>72 h	OECD 201, 2006	MBL<br>1.4x	3<br>10^5 cells/mL	CTR; 1.5 – 11.3	CTR; 3.0 – 22.6	CTR; 3.0 – 22.6
	Growth rate<br>72 h	OECD 201, 2006	MBL<br>1.4x	3<br>10^5 cells/mL	CTR; 1.5 – 11.3	CTR; 3.0 – 22.6	CTR; 3.0 – 22.6
Daphnia magna	Mortality<br>48 h	OECD 202, 2004	ASTM<br>1.2x	4<br>5	CTR; 0.74 – 1.84	CTR; 2.68 – 5.56	CTR; 2.68 – 5.56

	Feeding 24 h	Allen et al., 1995	ASTM 1.3x	4 5	CTR; 0.21 – 1.32	CTR; 1.92 – 5.47	CTR; 1.96 – 5.61
	Somatic growth 72 h	Burns, 2000	ASTM 1.2x	10 1	CTR; 0.33 - 1.0	CTR; 1.06 - 3.79	CTR; 1.05 - 3.77
Brachionus calyciflorus	Mortality 24 h	RoTox Kit F® Acute	ASTM 1.2x	5 5	CTR; 3.19 – 9.53	CTR; 3.19 – 9.53	CTR; 3.19 – 9.53
	Reproduction 48 h	RoTox Kit F® Chronic	ASTM 1.2x	5 1	CTR; 1.07 – 2.21	CTR; 1.07 – 2.66	CTR; 1.07 – 3.83
Hydra viridis-sima	Mortality 96 h	Trottier et al., 1997; Quinn et al., 2007	Standard medium 1.2x	6 1	CTR; 0.58 – 1.42	CTR; 1.06 – 3.92	CTR; 1.09 – 2.27
	Malformations 96 h	Trottier et al., 1997; Quinn et al., 2007	Standard medium 1.2x	6 1	CTR; 0.27 – 0.82	CTR; 1.06 – 3.92	CTR; 2.27 – 5.50
Danio rerio	Mortality 96 h	OECD 236, 2013	Zebrafish water 1.4x	10 1	CTR; 0.45 – 4.73	CTR; 1.69 – 24.9	CTR; 4.18 – 43.7
	Length 96 h	OECD 236, 2013	Zebrafish water 1.4x	10 1	CTR; 0.45 – 4.73	CTR; 1.69 – 24.9	CTR; 4.18 – 43.7

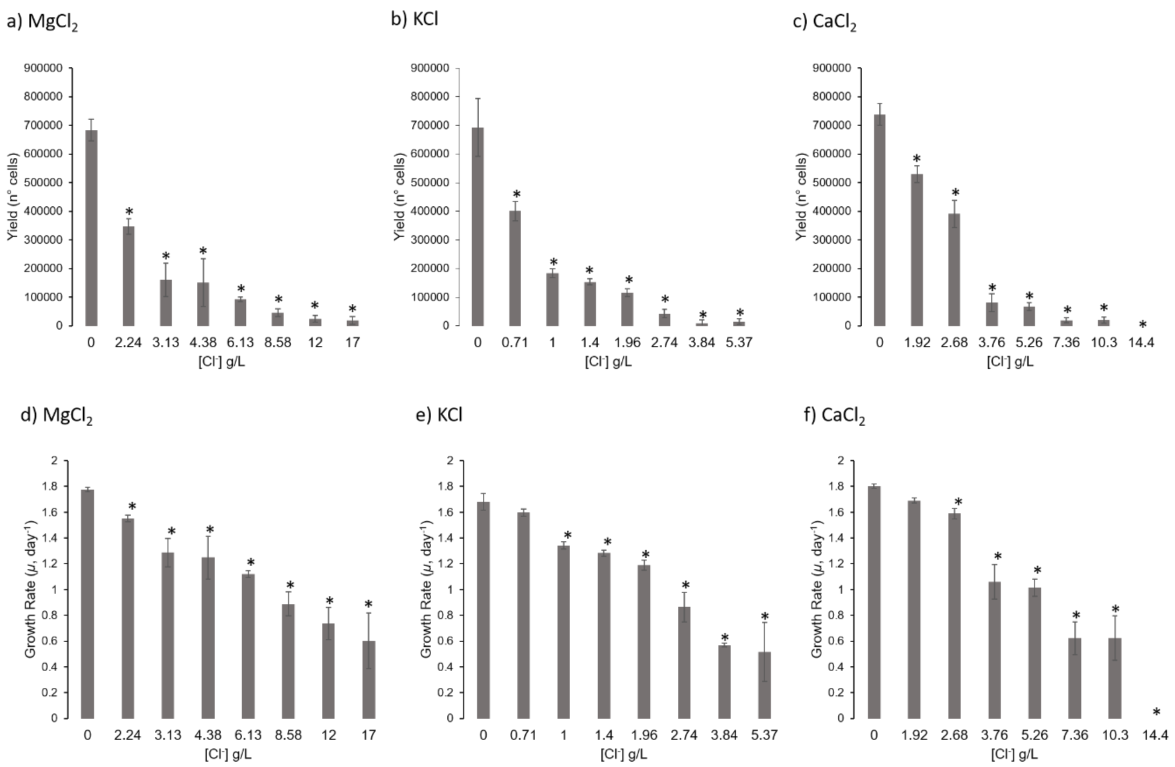

Figure S1: Yield (number of cells; graphics a to c) and average growth rate ($\mu.day^{-1}$; graphics d to e) of the green unicellular algae *Raphidocelis subcapitata* after 72 h of exposure to increased concentrations of three salts (magnesium chloride – $MgCl_2$; potassium chloride -KCl; and calcium chloride - $CaCl_2$). Concentrations are expressed in chloride (Cl^-; g/L). Vertical bars correspond to the standard deviation. *indicates statistically different in relation to control conditions after Dunnett's ($p<0.05$).

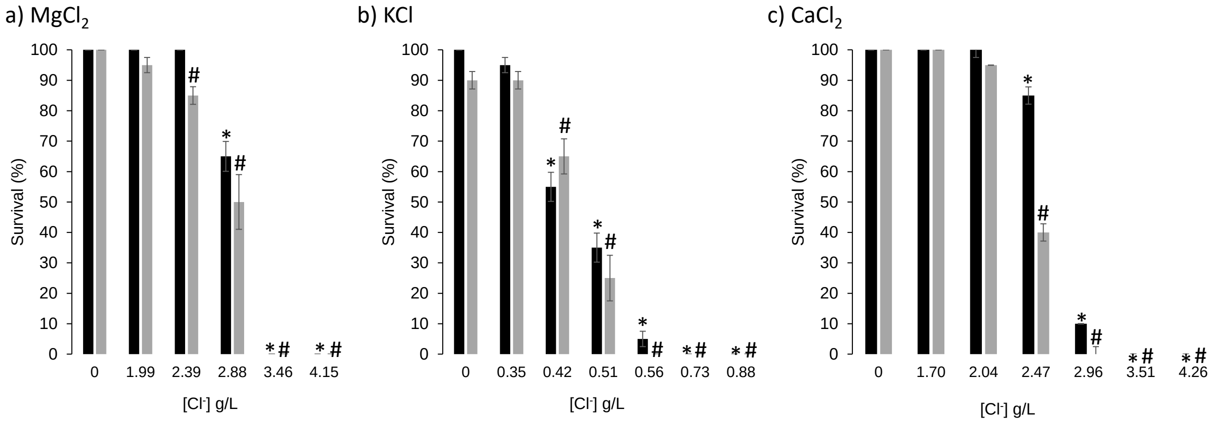

Supplementary Figure S2: Survival percentage of *Daphnia magna* after 24 h (black bars) and 48 h (gray bars) of exposure to increased concentrations of three salts (magnesium chloride – $MgCl_2$; potassium chloride -KCl; and calcium chloride - $CaCl_2$). Concentrations are expressed in chloride (Cl^-; g/L). Vertical bars correspond to the standard deviation. * and # indicates statistically different in relation to control conditions after 24h and 48h of exposure, respectively (Dunn's, $p<0.05$).

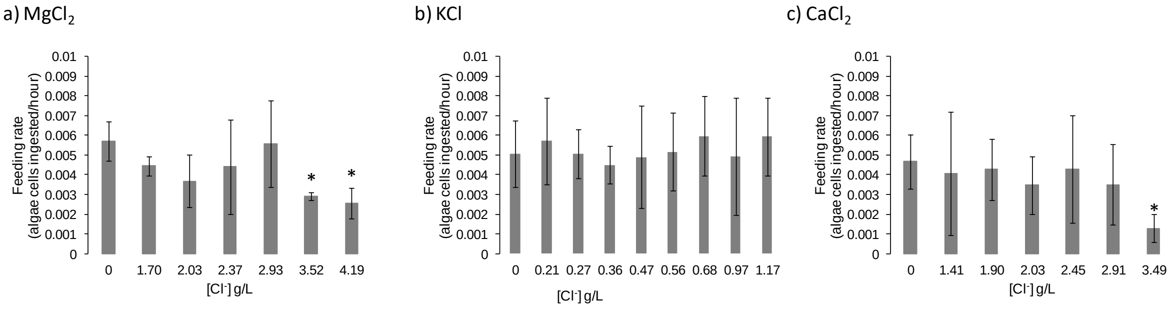

Supplementary Figure S3: Feeding rates (algae cells ingested/hour) of *Daphnia magna* after 24 h of exposure to increased concentrations of three salts (magnesium chloride – MgCl2; potassium chloride -KCl; and calcium chloride - CaCl2). Concentrations are expressed in chloride (Cl^-; g/L). Vertical bars correspond to the standard deviation. *indicates statistically different in relation to control conditions (Dunnett's, $p<0.05$).

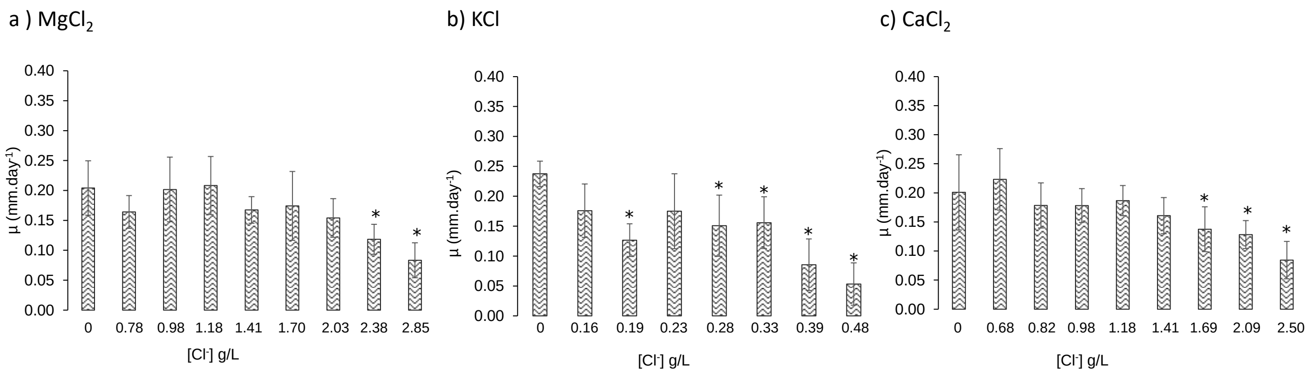

Supplementary Figure S4: Daily growth rate (μ; mm.day^{-1}) of *Daphnia magna* after 72 h of exposure to increased concentrations of three salts (magnesium chloride – $MgCl_2$; potassium chloride -KCl; and calcium chloride - $CaCl_2$). Concentrations are expressed in chloride (Cl^-; g/L). Vertical bars correspond to the standard deviation. *indicates statistically different in relation to control conditions (Dunnett's, $p<0.05$).

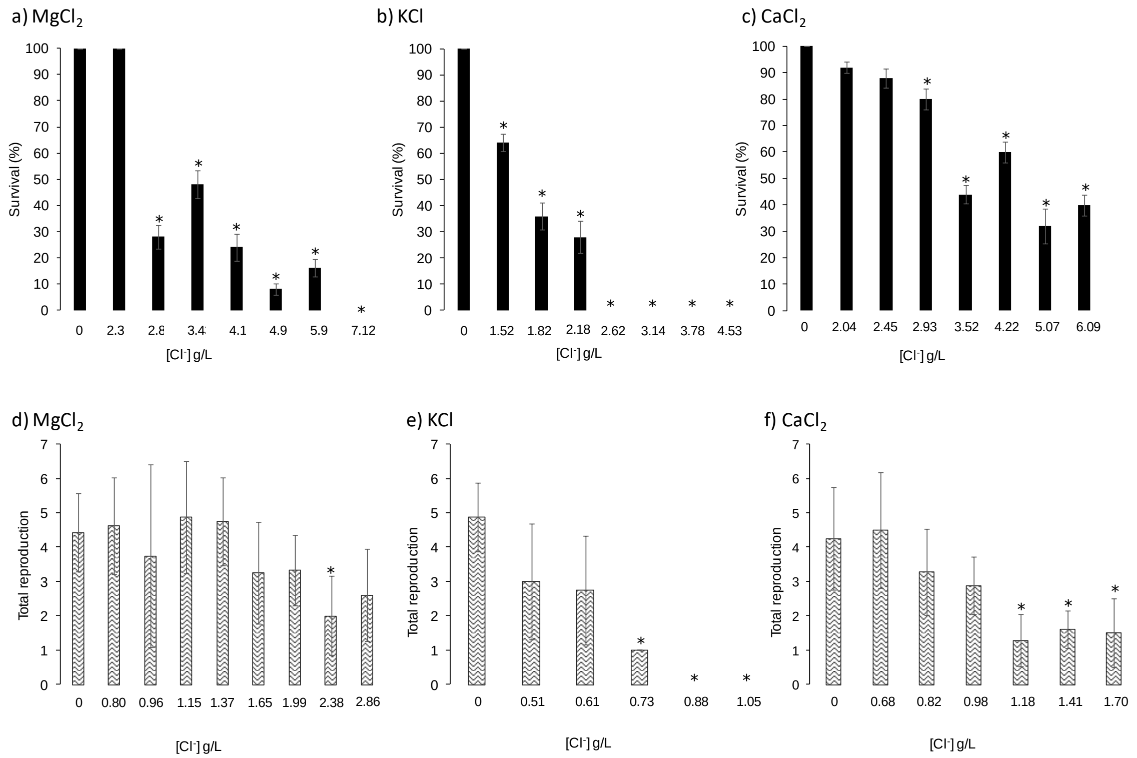

Supplementary Figure S5: Survival percentage (a trough c) and total reproduction (as the number of released offspring; d trough f) of the freshwater rotifer *Brachionus calyciflorus* after 24 h and 48 h of exposure, respectively, to increased concentrations of three salts (magnesium chloride – $MgCl_2$; potassium chloride -KCl; and calcium chloride - $CaCl_2$). Concentrations are expressed in chloride (Cl^-; g/L). Vertical bars correspond to the standard deviation. *indicates statistically different in relation to control conditions (Dunn's, $p<0.05$).

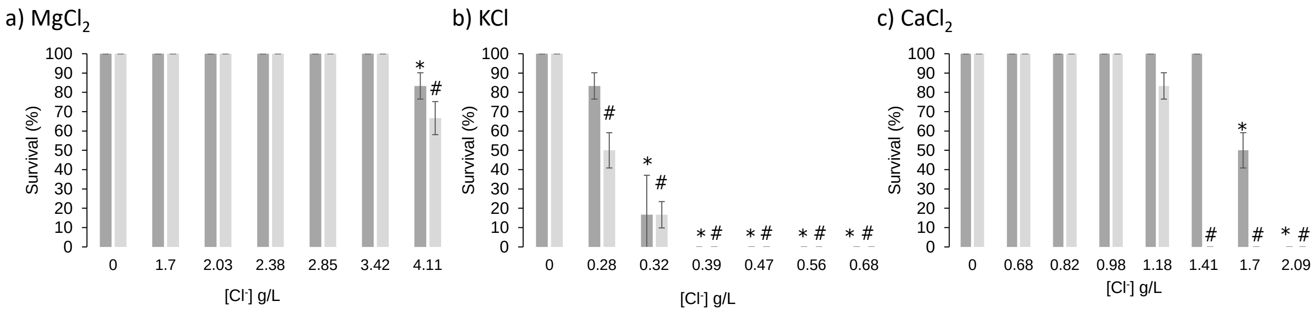

Supplementary Figure S6: Survival percentage of the cnidarian *Hydra viridissima* after 48 h and 96 h of exposure (dark and light gray bars, respectively), to increased concentrations of three salts (magnesium chloride – $MgCl_2$; potassium chloride - KCl; and calcium chloride - $CaCl_2$). Concentrations are expressed in chloride (Cl^-; g/L). Vertical bars correspond to the standard deviation. * and # indicates statistically different in relation to control conditions after each exposure period 48h or 96h, respectively (Dunn's, $p<0.05$).

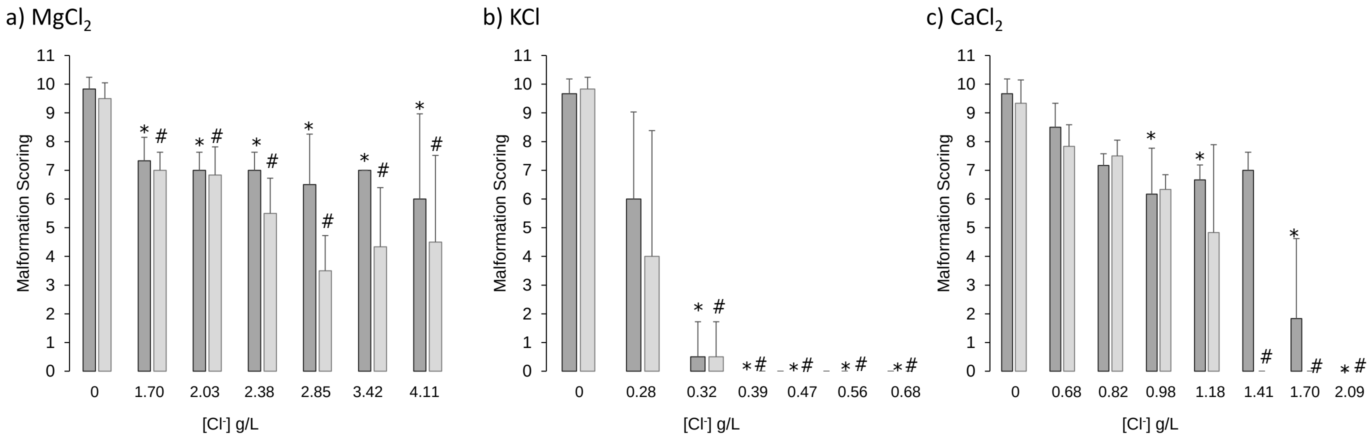

Supplementary Figure S7: Malformation scoring (according to Wilby, 1988) attributed to the cnidarian *Hydra viridissima* after 48 h and 96 h of exposure (pattern bars and full bars, respectively), to increased concentrations of three salts (magnesium chloride – $MgCl_2$; potassium chloride -KCl; and calcium chloride - $CaCl_2$). Concentrations are expressed in chloride (Cl^-; g/L). Vertical bars correspond to the standard deviation. * and # indicates statistically different in relation to control conditions after each exposure period 48h or 96h, respectively (Dunn's, $p<0.05$).

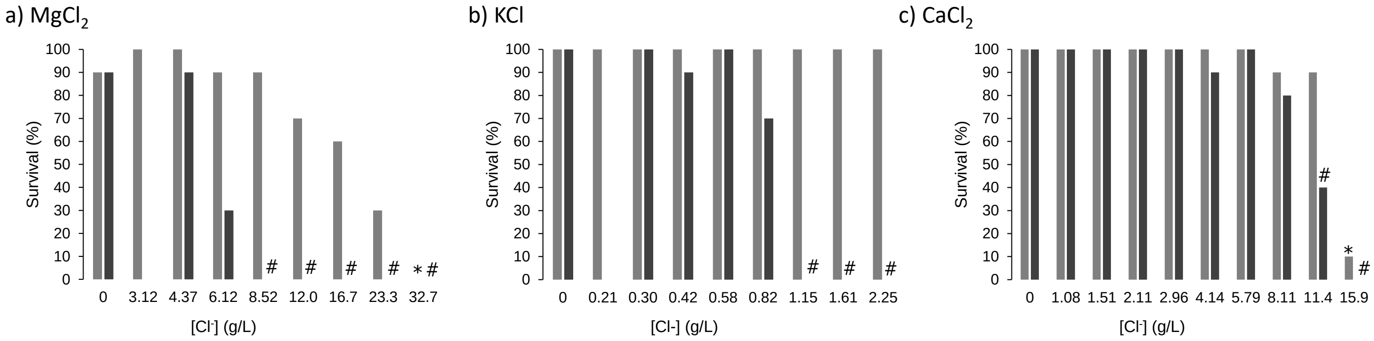

Supplementary Figure S8: Survival percentage of *Danio rerio* embryos exposed for 48 h (black bars) and 96 h (gray bars) to increased concentrations of three salts (magnesium chloride – $MgCl_2$; potassium chloride -KCl; and calcium chloride - $CaCl_2$). Concentrations are expressed in chloride (Cl^-; g/L). * and # indicates statistically different in relation to control conditions after each exposure period 48h or 96h, respectively (Dunn's, $p<0.05$).

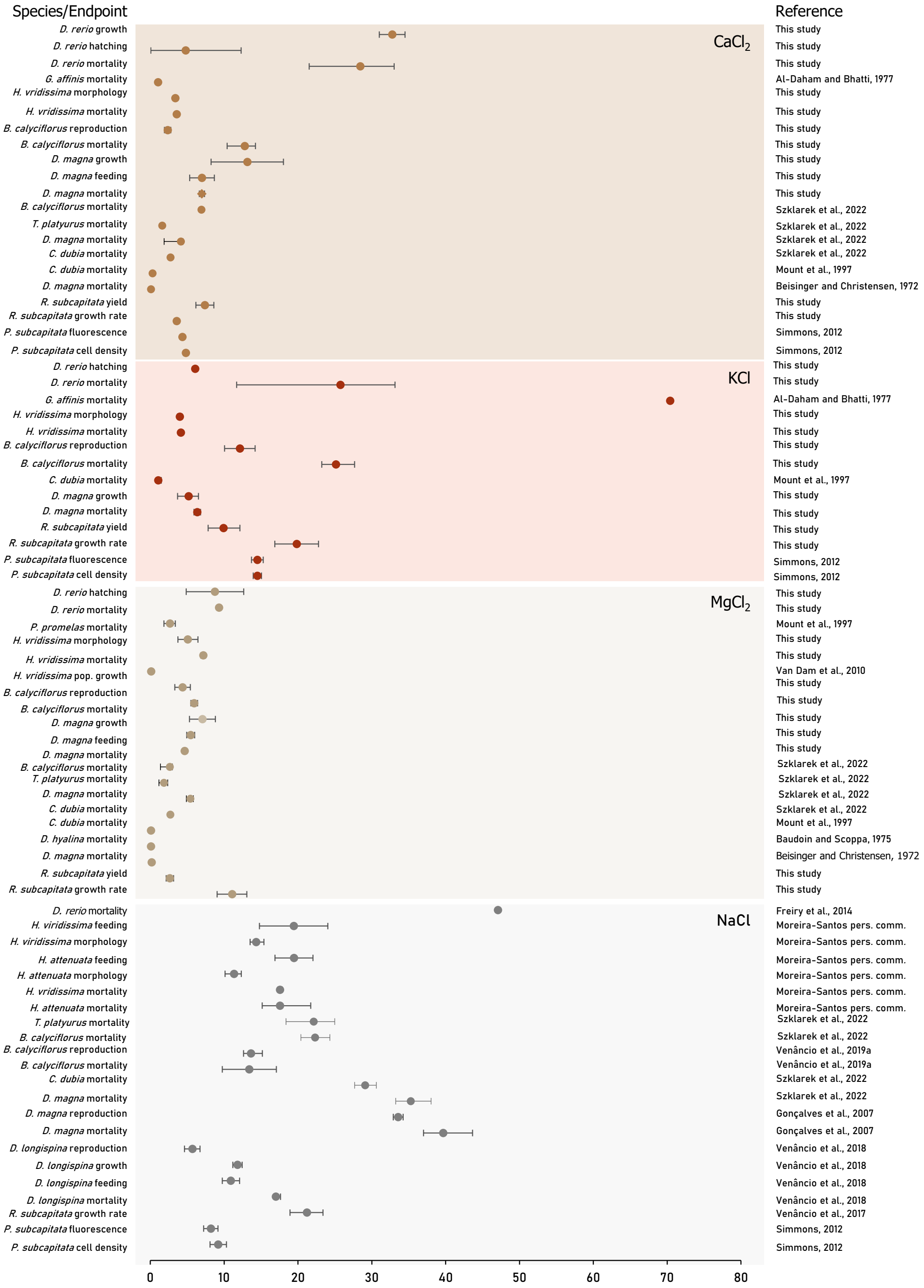

Supplementary Figure S9: Comparison of the median lethal and sublethal concentrations of the salts sodium chloride (NaCl), magnesium chloride ($MgCl_2$) , potassium chloride (KCl), and calcium chloride ($CaCl_2$), expressed in molarity of the respective cation, obtained in this study and collected from previous studies.

Supplementary Table S2: Range of values for the parameters measured at the beginning and end of each assay (highest and lowest pH, conductivity, and dissolved oxygen) for each of the three tested salts (magnesium chloride – $MgCl_2$, calcium chloride – $CaCl_2$, and potassium chloride – KCl).

	$MgCl_2$						$CaCl_2$						KCl					
Species endpoint	pH		Cond (mS/cm)		O_2 (mg/L)		pH		Cond (mScm)		O_2 (mg/L)		pH		Cond (mScm)		O_2 (mg/L)	
	start	end	start	end	start	end	start	end	start	end	start	end	start	end	start	end	start	end
Raphidocelis subcapitata 72-h growth rate assay	8.22-8.27	8.14-8.07	3.37-17.7	3.65-18.5	8.14-8.19	8.21-8.33	7.99-8.07	8.03-8.09	5.53-26.1	5.94-26.7	7.87-7.83	8.03-7.81	8.02-8.04	8.11-8.18	3.91-19.2	5.44-19.9	8.13-8.21	8.44-8.57
Daphnia magna 48-h mortality assay	8.26-8.59	8.50-8.61	1.10-2.09	1.10-2.14	8.21-8.13	8.19-8.09	7.92-7.89	8.06-8.15	4.87-7.70	5.78-7.86	8.22-8.34	8.17-8.05	7.80-7.93	7.83-7.91	1.06-2.06	1.03-2.09	8.22-8.17	8.18-8.02
D. magna 24-h feeding assay	7.89-7.96	8.06-8.14	2.33-5.30	2.52-5.47	8.13-8.22	8.08-8.17	7.66-7.78	7.87-7.81	3.36-7.60	3.40-7.65	8.16-8.25	8.17-8.03	7.82-7.91	8.03-8.11	1.48-3.40	1.54-3.36	8.22-8.28	8.08-8.17
D. magna 72-h growth assay	8.26-7.26	8.36-7.26	1.87-5.17	2.10-5.18	8.02-7.88	7.97-7.72	8.41-7.83	8.36-7.82	1.34-5.81	1.60-5.44	8.14-8.21	8.05-8.11	8.11-8.05	8.16-8.02	2.09-4.96	2.08-5.01	8.12-8.21	8.17-8.24
Brachionus calyciflorus 24-h mortality assay	8.07-7.83	8.77-8.12	3.50-9.16	3.48-9.21	8.54-8.11	8.56-7.85	7.63-7.55	7.95-7.77	4.99-12.7	4.84-12.8	8.53-8.01	8.03-7.77	8.35-8.28	8.14-8.35	5.86-16.2	5.79-16.4	8.96-7.71	8.11-7.58
B. calyciflorus 48-h reproduction assay	7.86-7.95	8.14-8.30	1.44-3.63	1.62-3.68	8.33-8.27	8.14-8.21	7.77-7.83	8.03-7.97	2.17-3.88	2.33-4.02	8.37-8.22	8.09-7.89	8.31-8.36	8.22-8.28	2.76-4.36	2.88-4.50	7.99-8.09	7.81-8.02-
Hydra viridissima 96-h mortality assay	6.04-5.98	6.12-6.27	2.30-5.38	2.35-5.47	8.21-7.49	7.61-7.11	6.79-6.91	7.03-6.98	1.76-5.35	1.93-5.53	8.22-7.89	8.03-7.79	5.83-5.90	5.91-6.05	3.86-7.81	3.87-7.92	7.98-7.73	7.11-7.74
H. viridissima 96-h malformation assay	7.03-6.84	6.99-6.54	2.14-5.00	2.29-5.27	8.17-8.24	8.14-8.08	6.79-6.91	7.03-6.98	1.76-5.35	1.93-5.53	8.22-7.89	8.03-7.79	6.91-6.37	6.87-6.54	1.06-1.94	1.21-2.06	8.03-7.87	7.99-7.63
Danio rerio 96-h mortality and malformation assay	7.66-7.53	7.56-7.62	2.89-15.3	3.12-15.6	8.03-8.11	7.98-8.05	7.72-7.48	7.75-7.56	3.26-29.7	3.71-30.3	8.27-8.16	8.22-8.04	7.83-7.79	7.71-7.66	2.13-8.54	2.18-8.56	8.17-8.11	8.08-7.99
